# Stimulator Selection in SSVEP-Based Spatial Selective Attention Study

**DOI:** 10.1155/2016/6410718

**Published:** 2016-12-01

**Authors:** Songyun Xie, Chang Liu, Klaus Obermayer, Fangshi Zhu, Linan Wang, Xinzhou Xie, Wei Wang

**Affiliations:** ^1^School of Electronics and Information, Northwestern Polytechnical University, Xi'an, China; ^2^School of Electronic Engineering and Computer Science, Technical University of Berlin, Berlin, Germany; ^3^Department of Electrical and Computer Engineering, University of Houston, Houston, TX, USA; ^4^AVIC Shenyang Aircraft Design & Research Institute, Shenyang, China

## Abstract

Steady-State Visual Evoked Potentials (SSVEPs) are widely used in spatial selective attention. In this process the two kinds of visual simulators, Light Emitting Diode (LED) and Liquid Crystal Display (LCD), are commonly used to evoke SSVEP. In this paper, the differences of SSVEP caused by these two stimulators in the study of spatial selective attention were investigated. Results indicated that LED could stimulate strong SSVEP component on occipital lobe, and the frequency of evoked SSVEP had high precision and wide range as compared to LCD. Moreover a significant difference between noticed and unnoticed frequencies in spectrum was observed whereas in LCD mode this difference was limited and selectable frequencies were also limited. Our experimental finding suggested that average classification accuracies among all the test subjects in our experiments were 0.938 and 0.853 in LED and LCD mode, respectively. These results indicate that LED simulator is appropriate for evoking the SSVEP for the study of spatial selective attention.

## 1. Introduction 

Attention is the cognitive process of selectively concentrating on one area while ignoring others [1]. Visual evoked potential provides a flexible way to study the visual cognitive process [2–4]. The Steady-State Visual Evoked Potential (SSVEP) is the response of the brain to a stimulus flickering at a constant frequency [4, 5]. SSVEP is recorded as a continuous oscillating with the stimulus frequency from the posterior scalp contra lateral [6–11]. The amplitude of SSVEP is enhanced when subjects' attention is cued to the stimulus. This contralateral distribution of the oscillation and the amplitude difference are efficient features to investigate visual spatial selective attention [12], and this selective feature has been applied in several researches as a measurement method such as the study of the binocular rivalry by Zhang et al. [13] and Wang et al. [14]

The SSVEP can be evoked by different flickers, such as Liquid Crystal Display (LCD), Light Emitting Diode (LED), and Cathode Ray Tube (CRT). The comparison suggested that CRT screen produced more visual fatigue than LCD [15] while there is no significant difference in SSVEP evoked signal by LCD and CRT [15]. Therefore, CRT has seldom been used as stimulator to evoke SSVEP recently whereas LCD devices were used widely; they are easily available in various pattern, but the flickering frequency adjustment is not flexible because the frequencies were limited by the refresh rate of the monitor and the target frequency could not be more than half of the refresh rate [16], whereas LED has many advantages over LCD [17]. Firstly, LED stimulator could evoke strong SSVEP in parietal and occipital cortex. Secondly, LED stimulator decreases the unrelated interference. Finally, LED flicker frequency is much flexible in both range and accuracy [18]; for example, it could evoke SSVEP in a frequency range of 1–90 Hz [17].

Since both the devices can evoke the SSVEP, the differences in SSEVP results from different simulation environments have been investigated by several researchers like the impact of stimulators in the study of brain computer interface [15, 17, 19, 20]. Wu et al. suggested that the differences of SSVEP were highly related to the frequency spectrum differences of the flickers used in the BCI system [17]. Cecotti et al. found that the quality of the stimuli is an important criterion for obtaining reliable SSVEP-BCIs and they proposed a new strategy based on the refresh rate of LCD screen for synchronizing the visual stimuli, which became more sensible for complex BCIs than LED based stimulators. Zhu et al. showed that the choice of properties of the used stimuli could affect the performance, safety, and comfort of an SSVEP-based BCI. Moreover, improvements in the stimuli process can enhance the SSVEP and SNR, simplify the signal processing, and assist in using more targets, preventing the attention loss and enabling BCI autonomous operation [19]. Thielen et al. investigated the complicated spectrums for the flickers [20]. Visual stimulation with pseudorandom bit-sequences evokes specific Broad-Band Visually Evoked Potentials (BBVEPs) that can be reliably used in BCI for high-speed communication in speller applications [20]. However, there is little information available in literature about the impact of stimuli on the studies of the spatial selective attention or its applications. In this paper, experimental results were presented to show that the differences of SSVEP were evoked by two different stimulators. The comparisons were carried out in the analysis of spectrum as well as the accuracy of classification based on two classical paradigms of spatial selective attention proposed by Morgan et al. [1] and Müller et al. [21] with slight changes, like the stimulating frequencies being changed to 8 Hz and 12 Hz in both LCD and LED condition for the contradistinctive reason. Both stimulators are proved to evoke the SSVEP successfully. In order to investigate the difference between the LED and LCD stimuli, the frequencies need to be identical. In this paper, the LCD stimulators' frequencies were set to 8 Hz and 12 Hz, since Morgan et al. proposed the frequencies 8.6 Hz and 12 Hz in their research paper; for comparison LED stimuli frequencies were also set to 8 Hz and 12 Hz, unlike 20.8 Hz and 27.8 Hz in the one proposed by Müller et al.

## 2. Methods and Arrangements

### 2.1. Frequency Selection

In the study of spatial selective attention, the stimulating frequencies must ensure that the responses are unique and strong enough to get the better pattern of results. Aforementioned studies showed that SSVEP would be weak if the stimulus frequency was beyond the range of 5–30 Hz [19]. Additional requirement is that the stimulating frequencies cannot be harmonics or subharmonics from each other [19]. These frequencies should not be selected within 15–25 Hz to avoid the risk of inducing photoepileptic seizures. Previous studies demonstrated that the evoked SSVEP signals were stronger in low frequency [22] and 8 Hz and 12 Hz caused less photosensitive responses than the frequencies in 15–25 Hz [23]. Thus, flickers with a frequency pair of 8 Hz and 12 Hz were selected in this work for the contradistinctive reason. In order to select optimal stimulator in the study of visual spatial selective attention, the efficiency of the stimulator should be explored. The frequency of LCD stimulus is limited by its refresh rate. But LED's frequency is flexible. In order to validate the advantages of LED stimulator in both range (can be set to higher frequency) and accuracy (0.1 Hz in the additional experiment), flickering phenomena in LED were also observed in the frequency pair of 20.8 Hz and 27.8 Hz.

### 2.2. Experiment and Paradigm

In this work, we concentrated on the stimulator selection in SSVEP-based spatial selective attention study. Therefore, the optimal paradigms of LED and LCD were selected, respectively. LED stimulator was presented as LED array in four flickering pattern; one of them was selected as target to enhance subject's attention. This is a classical paradigm to evoke SSVEP by LED stimulator in the study of spatial selective attention [21]. Moreover, LCD stimulator was presented as flickering square backgrounds with different characters on monitor. “5” was selected as target to enhance the subject's attention. Because of the flexibility in LCD, there are many ways to present the LCD stimulation [19]. Therefore, the LCD paradigm which makes subject more comfortable and focused was selected for the experiment.

#### 2.2.1. LCD Arrangement

**Figure 1:**
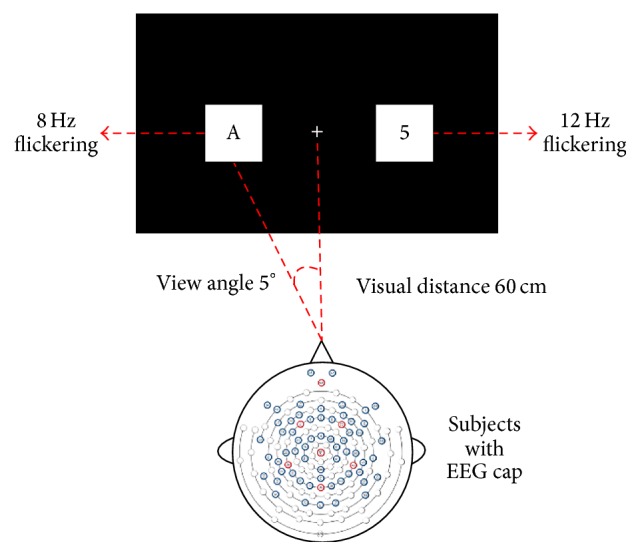
Dual visual field stimulus paradigm by LCD. Subjects monitored the character sequence in one visual field while ignoring the contralateral sequence. Square backgrounds were flickered at 8 Hz in the left field and 12 Hz in the right, which was implemented by two videos of characters in different frequencies. The distance between the monitor and subjects was 60 cm while the view angle was 5 degrees. The subjects were required to press a button as soon as they detect the target “5” to enhance their focus.

A classical left-right dual visual field stimulus paradigm was presented to evoke SSVEP by LCD [1], which is shown in Figure 1.

Two videos of characters generated in each trial were implemented by E-prime 2.0 (Psychology Software Tools, Inc., version 2.0.8.73). Subjects were directed to sit comfortably in a shielded chamber during the experiment and stared at visual stimulus presented on the LCD 60 cm in front of him/her.

#### 2.2.2. LED Arrangement

**Figure 2:**
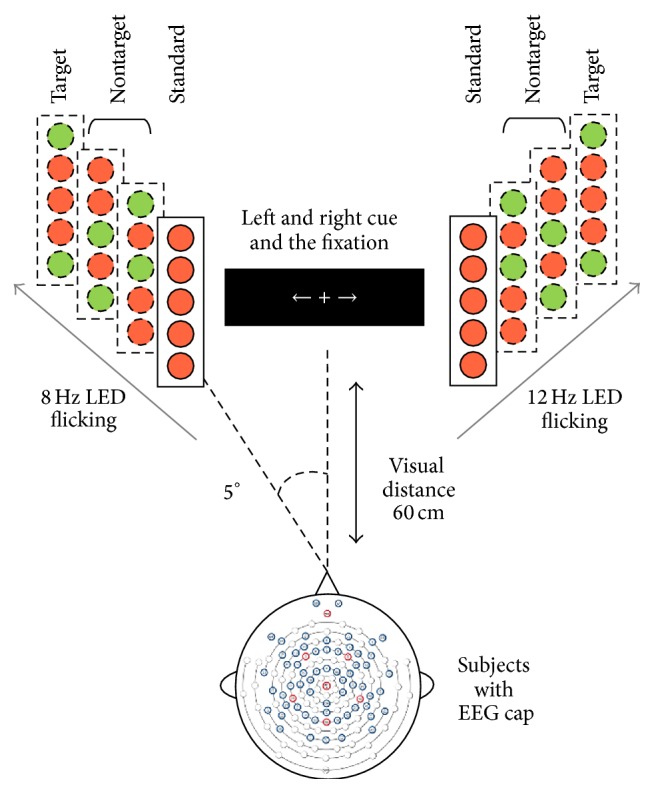
Dual visual field stimulus paradigm by LED (Miaoqi Electronic, BQ-3401UGC, 505 nm~530 nm; 6000–8000 mcd). The four bars on each side showed the four possible color configurations for each bar. A red circle represented a red LED while the green one represented a green LED. Cue and fixation “+” were displayed by monitor. The distance between the monitor and subjects was 60 cm while the view angle was 5 degrees. Frequency and pattern of LED flicker were controlled by FPGA (CYCLONE IV E, EP4CE15F17C8N) and GRRRG pattern (R means red, G means green) was regarded as the target and subjects were required to push a button once it appears, in order to enhance their focus.

Similar to LCD, in LED experiment, a left-right dual visual field stimulus paradigm was selected based on the classical one [21]. In order to compare with LCD experiment, the same flickering frequencies were adopted. The paradigm is shown in Figure 2.

The flicking LEDs form different patterns were RRRRR or GRGRR and so forth (R-red, G-green). In a trial, each pattern lasted for 20 periods. The appearing probabilities of each pattern were 70% for RRRRR and 10% for GRGRR, RRGRG, and GRRRG, respectively.

#### 2.2.3. Paradigm

**Figure 3:**
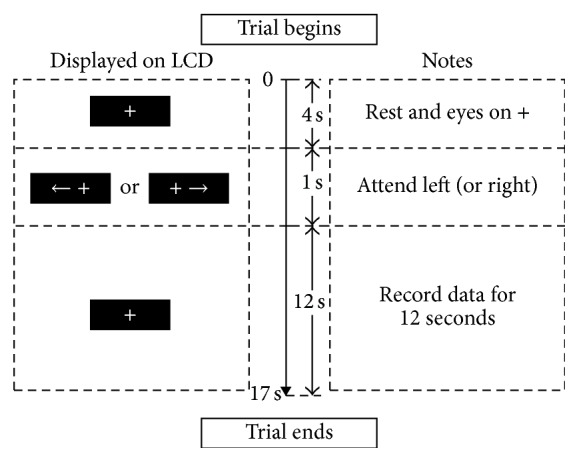
Design of the trial. At the beginning of each trial, an arrow will appear near the crossing, indicating which visual field (left/right) he/she should attend. Sequences and the flickering lasted 12 s in each trial.

The target in LCD experiment was character “5,” while the target in in LED experiment was pattern “GRRRG.” At the beginning of each trial, a fixation “+” was shown on the screen; then an arrow will appear near the crossing, indicating which visual field the subject should attend, as shown in Figure 3. Subjects were required to press a button as soon as they see the target to enhance the focus [15, 24].

Sequences and the flickering lasted 12 s in each trial and there were total 20 trials (10 attend left, 10 right) in the experiment.

#### 2.2.4. An Additional Experiment

In order to investigate the efficiency of LED stimulus, additional experiment was added. This time, the flickering frequencies were changed to 20.8 Hz and 27.8 Hz, which could verify the range (can be set to higher frequency) and accuracy (0.1 Hz in the additional experiment) of the LED stimulator.

### 2.3. Data Acquisition

The recording room was shielded with a Faraday cage (Changzhou Leining Electromagnetic Shield Equipment Co., Ltd., GPQ_2_), shielding electromagnetic interference from 14 kHz to 10 GHz. Test equipment was GES 300 System (EGI product; sensor array: 64-channel adult-sized head cap; EEG acquisition software: Net Station; computer: PowerPC G5; amplifier: Net Amps 300). The sample rate was 250 Hz, and the filtering window was 0.3 to 100 Hz.

Data were collected from 8 healthy right-handed volunteers (6 males, 2 females; age: 22 ± 3 years) with normal (or corrected to normal) vision. Most of the processing was performed in Matlab (Version R2013a, MathWorks) and some preprocessing, such as filtering, segmentation, and reference electrode conversion, was executed by Net Station (Version 4.5.7, EGI).

### 2.4. Preprocessing

Among the three experiments, the EEG data were filtered through 1–40 Hz band-pass filter. Raw EEG data were segmented to 20 trials (10 attend left, 10 attend right) for each channel. Each epoch was carefully checked and the bad trial was rejected for further analysis, which contained the artifacts related to eye blink, eye movement, and EMG.

### 2.5. Frequency Analysis

Fast Fourier Transform (FFT) technique was adopted for the power spectrum analysis and the topological graph was plotted to show the differences of two types of SSVEP, evoked by LCD and LED, in the frequency domain.

### 2.6. Short SSVEP-Based Classification

**Figure 4:**
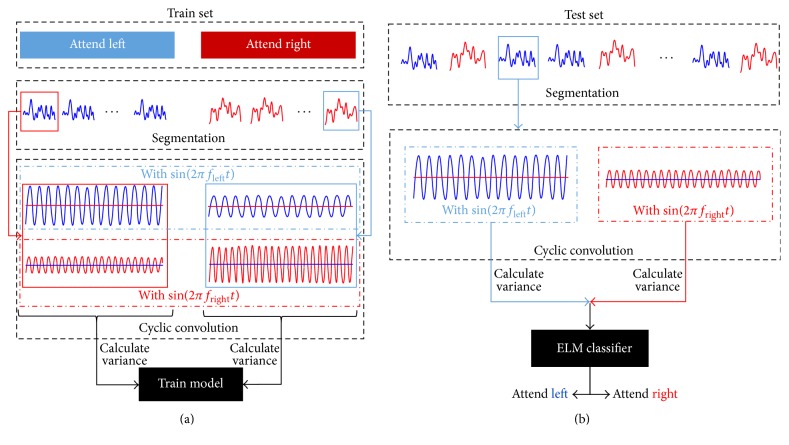
(a) The schematic diagram of the train algorithm. Frist, segment the raw data into small pieces (segmented EEG). Second, convolute each segmented EEG with sine signal in both two frequencies, respectively (short SSVEPs). Finally, calculate the short SSVEPs variance as the final feature and training the classifier. (b) The schematic diagram of the test algorithm.

In order to reduce the influence of the nontarget frequency on extracting the feature of SSVEP to a maximum extent, as shown in Figure 4, short SSVEP-based classification was selected. First, the EEG data was segmented into small pieces (lasts 3 s). Then, in order to extract EEG's feature on the flickering frequency [6], the segmented EEG was circular convoluted with the sine signals which have the same frequencies of the flickering stimuli in both sides, respectively, that is, the short SSVEPs. The final classification was based on the variances of the short SSVEPs. Extreme Learning Machine (ELM) was employed in classification [25]. The train-test ratio was 1 : 1 and selected randomly. The linear kernel function was selected after comparing the classification accuracies among different kernels.

## 3. Results

### 3.1. Frequency Analysis and Electrodes Chosen

**Figure 5:**
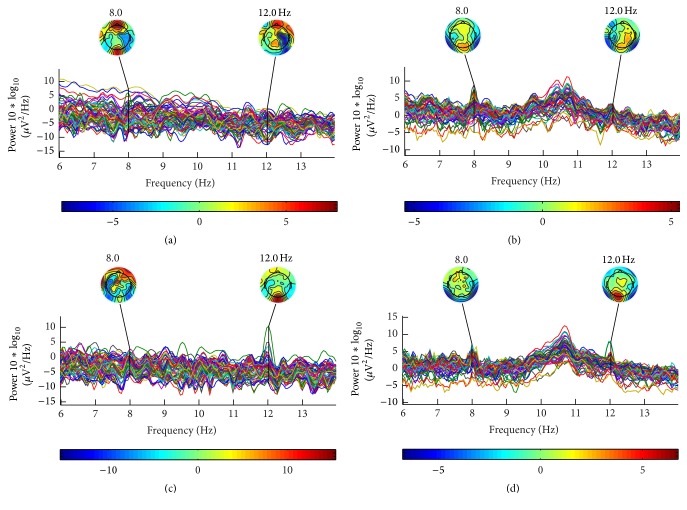
The result of PSD and topological graph. (a) PSD and topological graph of all channels at in LED mode when subject noticed 8 Hz; (b) PSD and topological graph of all channels in LCD mode when subject noticed 8 Hz; (c) PSD and topological graph of all channels in LED mode when subject noticed 12 Hz; (d) PSD and topological graph of all channels in LCD mode when subject noticed 12 Hz.

The frequency analysis results in the LED experiment and LCD experiment were shown in Figure 5. The pseudocolor was adopted to present the electrical activities in the topological graph; red represents the strong neural activities in that area, while blue represents the weak activities.  Figure 5(a) showed the results when the subject had attention on 8 Hz in LED mode while Figure 5(b) was in LCD mode; similarly, Figures 5(c) and 5(d) were those having attention on 12 Hz.

**Figure 6:**
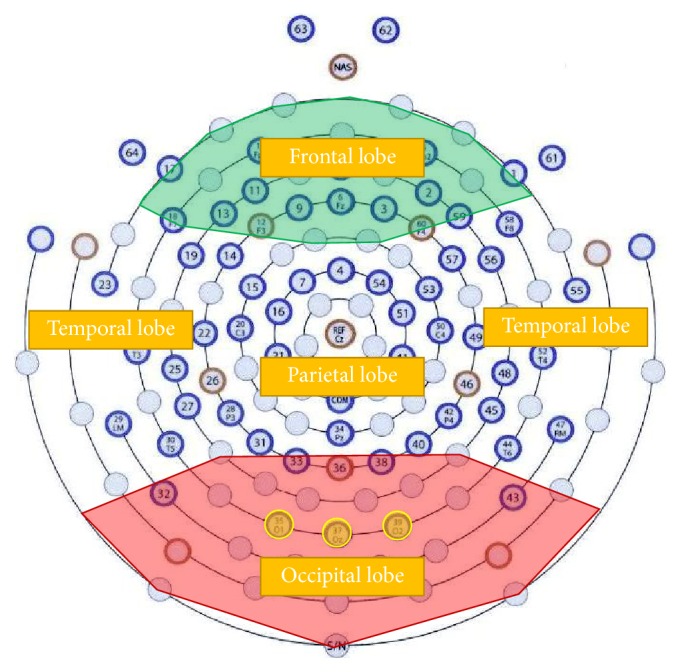
Position of the recording electrodes. Green indicates the frontal area, and red indicates the occipital area. The electrodes marked with yellow were selected after analysis.

According to Figure 5, both LED and LCD could successfully evoke SSVEP signals. However, the intensities for the evoked SSVEP at attention and inattention states were quite different among the different channels, especially on occipital area. Strong SSVEP had already been induced by LED, which was consistent with previous researches [13, 22, 26, 27]. In LCD mode, the results were similar. The most distinctive and prominent electrodes at the stimulating frequency were selected for further study in this paper, namely, O_1_ (CH35), O_z_ (CH37), and O_2_ (CH39) in Figure 6.

**Figure 7:**
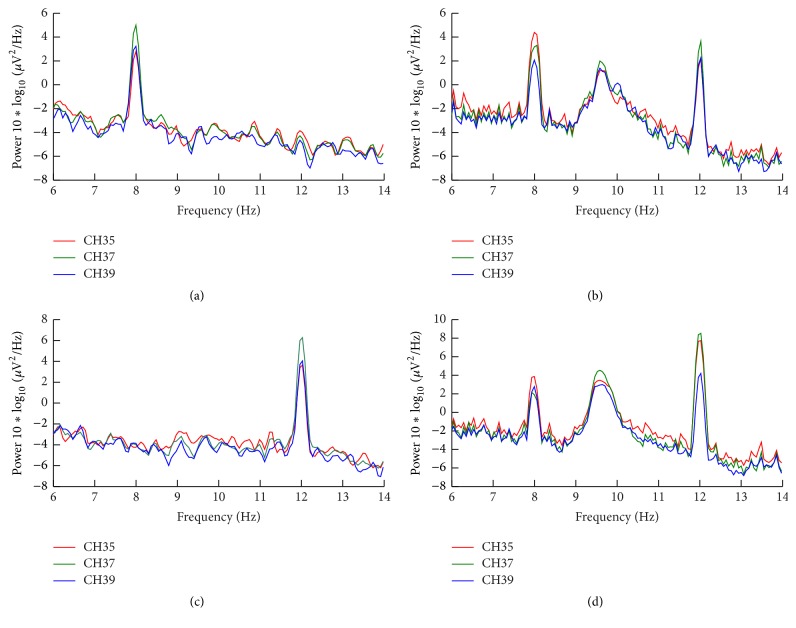
The result of PSD of O_1_, O_z_, and O_2_. (a) Averaged PSD in LED mode when subjects noticed 8 Hz; (b) averaged PSD in LCD mode when subjects noticed 8 Hz; (c) averaged PSD in LED mode when subjects noticed 12 Hz; (d) averaged PSD in LCD mode when subjects noticed 12 Hz. A strong frequency component around 10 Hz was observed, which was the interference of nontarget frequency (target frequency: 8 and 12 Hz in this situation). These results may be explained by gray to gray (GTG) and scanning of the monitor and cannot easily be eliminated [15, 19].

The PSD analysis of subjects was carried out on the results of O_1_ (CH35), O_z_ (CH37), and O_2 _(CH39), and the averaged results were shown in Figure 7. In LED mode (Figures 7(a) and 7(c)), higher power of EEG at the frequency where the subject was attentive was observed and the differences between the one of noticed frequency and others were significant. In LCD mode (Figures 7(b) and 7(d)), the amplitudes of evoked SSVEP on attention frequency and inattention frequency were almost the same, especially in Figure 7(b).

In order to explore the efficiency of LED mode, an additional experiment was added, in which the paradigm was the same as the paradigm shown in Figure 3 except for the flicking rates that were changed to 20.8 Hz on left side while they were changed to 27.8 Hz on the right, and these frequencies were in medium frequency band that were also in beta wave band [28, 29].

**Figure 8:**
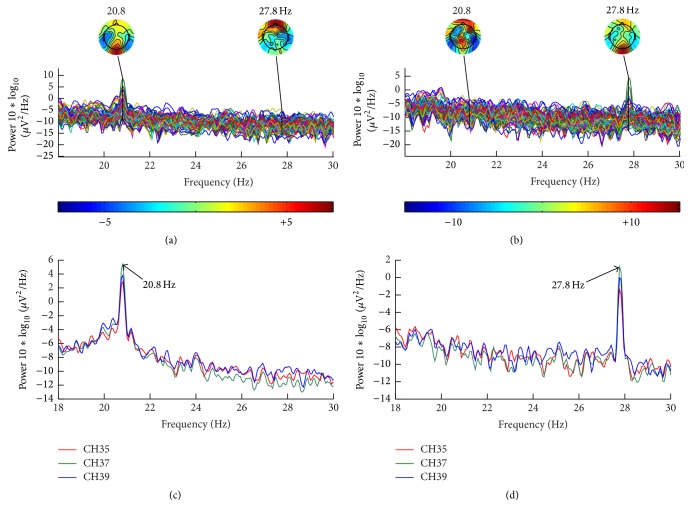
The result of PSD. (a) PSD of all channels when subject noticed 20.8 Hz; (b) PSD of all channels when subject noticed 27.8 Hz; (c) averaged PSD of O_1_, O_z_, and O_2_ among all subjects when they noticed 20.8 Hz; (d) averaged PSD of O_1_, O_z_, and O_2_ among all subjects when they noticed 27.8 Hz.

Figures 8(a) and 8(b) showed that there were significant differences in topological graph. Difference on occipital lobe on each subgraph was observed obviously, and this kind of nervous excitation showed that the strong SSVEP in LED paradigm has been stimulated. Figures 8(c) and 8(d) showed that there was much higher power of EEG at the frequency which the subject paid attention to and the differences among the different frequencies were significant. So, the results led to the conclusion that, in the LEDs mode, strong SSVEP in high frequency appeared in occipital lobe and the evoked frequencies were precisely; therefore, it was suitable to be used in spatial selective attention study especially for multiregion one.

### 3.2. Classification

**Figure 9:**
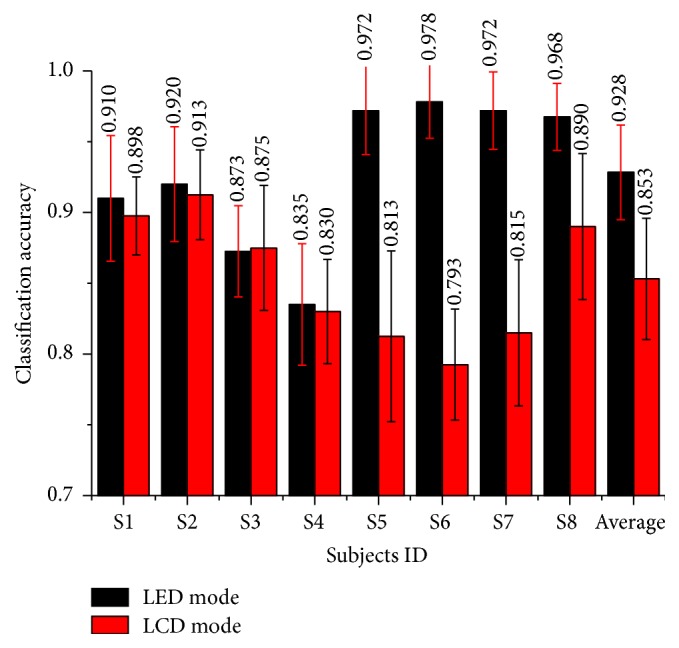
Classification accuracy of two models. Black bars represent the accuracy of LED mode while red bars represent the accuracy of LCD mode. Vertical lines give standard errors.

**Figure 10:**
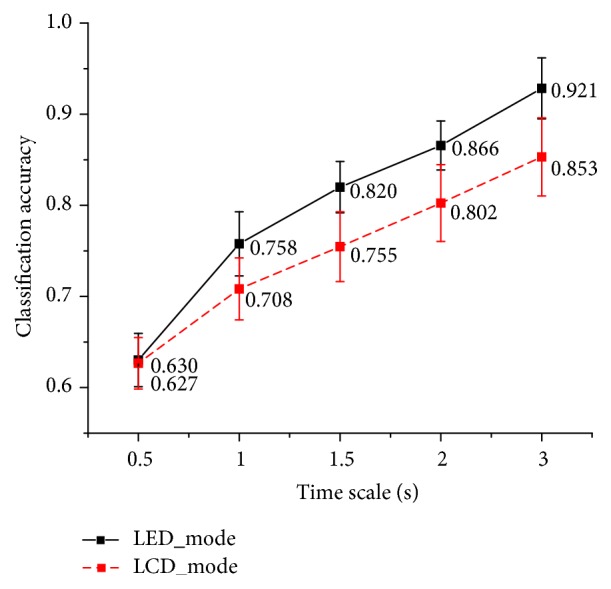
Classification accuracies of different modes among different time scales. The black solid line represents the accuracy of LED mode while the red dash line represents the accuracy of LCD mode. Vertical lines give standard errors.

The classification process has been carried out 10 times on each subject's data and the classification accuracies were shown in Figure 9. Results showed that there were higher classification accuracies (average rate = 0.928) for LED mode than LCD mode (average rate = 0.853) in the time scale of 3 s. The results for different time scales among all subjects were shown in Figure 10. Larger time scales would result in higher classification accuracies in both LED and LCD mode. And for all time scales, higher classification accuracies were observed in LED mode.

## 4. Discussion

### 4.1. Analysis of Spectrum

This comparison experiment between LCD and LED was carried out and SSVEP signals were both evoked by these two devices with flickers; still there are apparent spectrum frequency differences between attentive and inattentive states of subject; moreover the topological graph in LED mode suggests that the SSVEPs evoked by it have the strong discriminability, which is an important feature when studying the spatial selective attention. Consequently, it is possible to detect the attention distribution of the subject effectively by using LED stimulator.

Furthermore, in LCD mode, a strong frequency component around 10 Hz was observed. It was in alpha band and cannot be easily eliminated. However, in LED mode, this alpha component was not observed. Since the alpha waves are in close relationship with the degree of the attention (as pointed by Dewan [30] and Mulholland et al. [31], there was a significant inverse relationship between alpha wave and attention), these results might imply that LCD would result in less attention. In LCD mode, the monitor needs to be refreshed at given frequency and it has the gray to gray (GTG) response time (gray to gray is a response time of the monitor from one gray level to another gray level). These will be the reasons for the observed alpha component.

The result from the additional experiment demonstrated that the LED stimulator could evoke the SSVEP with high frequency precision, which makes it possible to divide the space into more subspaces. Beside this, the flexibility of LED stimulator in both range and accuracy were also tested in this part.

### 4.2. Accuracy of Classification

The results obtained from the classification accuracy graph proposed that the SSVEPs evoked by LED mode were easier to distinguish as compared to the LCD mode in all time scales from 0.5 s to 3 s which will contribute to the detection of the subject's selective attentiveness which enable us to use it in the selective attention as a measurement method with high accuracy.

## 5. Conclusion

In this paper, the responses of evoked SSVEP from two different stimulators were studied in detail. The comparison of the two different stimulators was carried out in the analysis of spectrum as well as the accuracy of classification based on two classical paradigms of spatial selective attention proposed by Morgan et al. and Müller et al. with slight modifications. Results showed that strong SSVEP components could be evoked in LED mode as compared to the LCD mode. The best region to acquire the SSVEP was occipital lobe for both modes. The flexibility in generating highly precise and the wide range of stimulating frequency are the advantages of LED mode.

Beside this, there is a significant difference between noticed and unnoticed frequency spectrum while in LCD mode this difference was relatively limited and it was difficult to change the stimulating frequencies. The classification accuracies among all subjects were 0.938 and 0.853 in LED mode and in LCD mode, respectively. This leads us to the conclusion that LED stimulator is an appropriate equipment to study the spatial selective attention and its applications.
